# Monocyte–Macrophage Lineage Cell Fusion

**DOI:** 10.3390/ijms23126553

**Published:** 2022-06-12

**Authors:** Malgorzata Kloc, Arijita Subuddhi, Ahmed Uosef, Jacek Z. Kubiak, Rafik M. Ghobrial

**Affiliations:** 1 Transplant Immununology, The Houston Methodist Research Institute, Houston, TX 77030, USA; asubuddhi@houstonmethodist.org (A.S.); auosef@houstonmethodist.org (A.U.); rmghobrial@houstonmethodist.org (R.M.G.); 2 Department of Surgery, The Houston Methodist Hospital, Houston, TX 77030, USA; 3 Department of Genetics, MD Anderson Cancer Center, The University of Texas, Houston, TX 78712, USA; 4 Dynamics and Mechanics of Epithelia Group, Faculty of Medicine, Institute of Genetics and Development of Rennes, University of Rennes, CNRS, UMR 6290, 35000 Rennes, France; jacek.kubiak@univ-rennes1.fr; 5 Laboratory of Molecular Oncology and Innovative Therapies, Department of Oncology, Military Institute of Medicine, 04-141 Warsaw, Poland

**Keywords:** cell fusion, syncytium, monocyte, macrophage, osteoclast, hematopoietic stem cells, giant cells, viral fusion, cell protrusions, podosomes, tumor-associated macrophages

## Abstract

Cell fusion (fusogenesis) occurs in natural and pathological conditions in prokaryotes and eukaryotes. Cells of monocyte–macrophage lineage are highly fusogenic. They create syncytial multinucleated giant cells (MGCs) such as osteoclasts (OCs), MGCs associated with the areas of infection/inflammation, and foreign body-induced giant cells (FBGCs). The fusion of monocytes/macrophages with tumor cells may promote cancer metastasis. We describe types and examples of monocyte–macrophage lineage cell fusion and the role of actin-based structures in cell fusion.

## 1. Monocyte–Macrophage Cell Lineage

**Figure 1:**
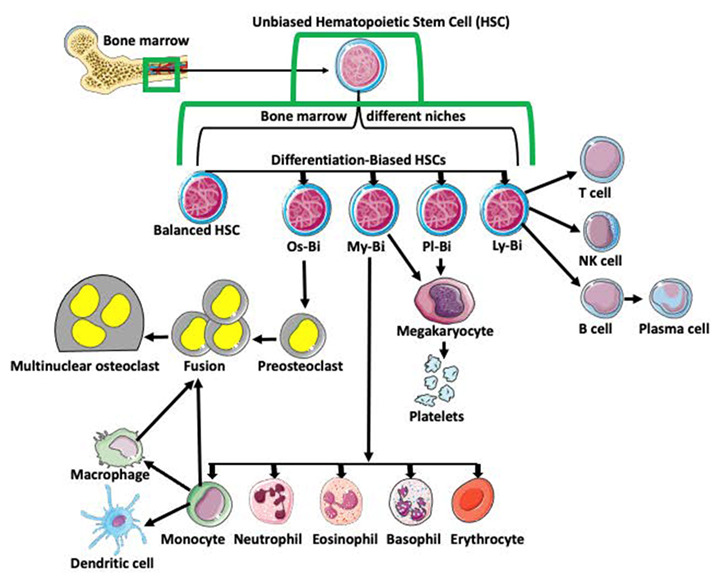
Differentiation of hematopoietic stem cells (HSCs). Multipotent and unbiased hematopoietic stem cells (HSCs) are derived from bone marrow. Depending on the niche they occupy in bone marrow and/or expression of certain genes, they become biased in their differentiation potential toward a specific cell lineage, such as osteoclast-biased (Os-Bi), myeloid-biased (My-Bi), platelet biased (Pl-Bi), and lymphoid-biased (Ly-Bi). Some HSCs have balanced differentiation potential and can develop into osteoclast, myeloid, platelet, and lymphoid lineage precursors. Os-Bi HSCs develop into preosteoclasts which, after fusion, create multinucleated osteoclasts. Osteoclasts can also derive from mature monocytes or macrophages. The My-Bi HSCs differentiate into neutrophils, eosinophils, basophils, erythrocytes, and monocytes forming dendritic cells and macrophages. Pl-Bi HSCs develop into megakaryocytes, which subsequently produce platelets. Megakaryocytes can also develop from My-Bi HSCs. Ly-Bi develop into T cells, NK cells, and B cells producing plasma cells.

**Figure 2:**
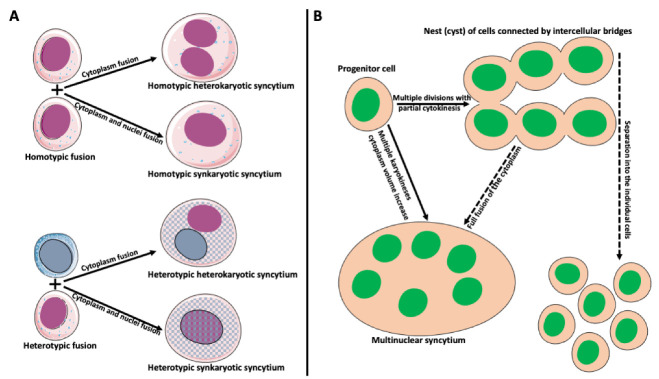
Types and origin of syncytia. (**A**) Syncytium derived from the fusion of identical cells is called homotypic syncytium. Homotypic fusion of cell cytoplasms creates homotypic heterokaryotic syncytium with multiple nuclei. Homotypic fusion of cell cytoplasms and nuclei creates homotypic synkaryotic syncytium. Syncytium derived from the fusion of different cell types is called heterotypic syncytium. Heterotypic fusion of only cell cytoplasms creates heterotypic heterokaryotic syncytium with multiple nuclei of different origins. Heterotypic fusion of cell cytoplasms and nuclei creates heterotypic synkaryotic syncytium. (**B**) Origin of syncytia during development. In some instances, progenitor cell divides multiple times with partial cytokinesis forming a group (called nest or cyst) of descendant cells connected by cytoplasmic bridges. Eventually, these cells either separate into individual cells (for example, in frog or mammalian oogenesis) or fuse to form multinuclear syncytium (for example, nurse cell syncytium in insect telotrophic ovary). In other instances, for example, during early embryogenesis in *Drosophila*, the nucleus of the progenitor cell divides multiple times, creating a multinuclear cell (syncytium), which eventually cellularizes into individual cells.

**Figure 3:**
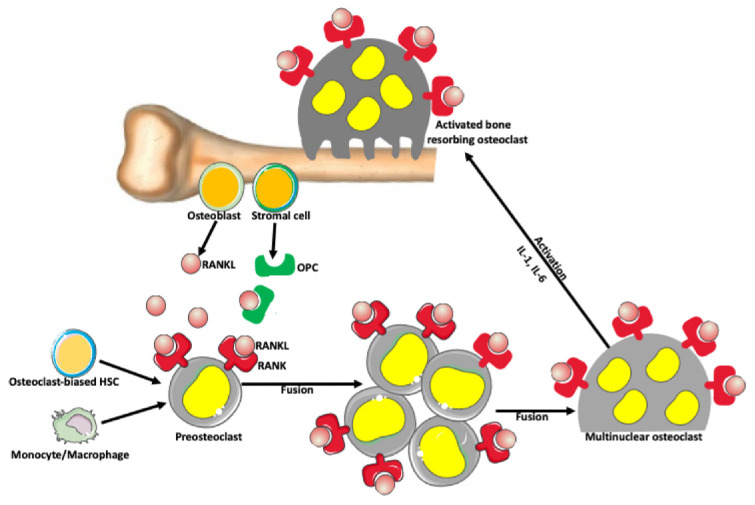
Osteoclast formation. There is a balance between bone-forming and bone-resorbing activities in normal conditions. The bone-resorbing cells’ osteoclasts form through the fusion of preosteoclasts, which are derived from monocytes/macrophages and/or osteoclast-biased hematopoietic stem cells (HSC). Osteoblasts and stromal cells in the bone produce a receptor activator of nuclear factor-kappa-Β ligand (RANKL), which belongs to a tumor necrosis factor family of proteins. RANKL binds to its receptor RANK expressed on the surface of preosteoclasts and osteoclasts, promoting fusion and formation of syncytial multinuclear osteoclasts. After further activation by various cytokines, mature osteoclasts acquire a bone-resorbing activity. Osteoblast and stromal cells also produce osteoprotegerin (OPC) that prevents excessive bone resorption by binding to and depleting RANKL. Thus, RANKL/OPC ratio determines bone resorption or bone formation.

**Figure 4:**
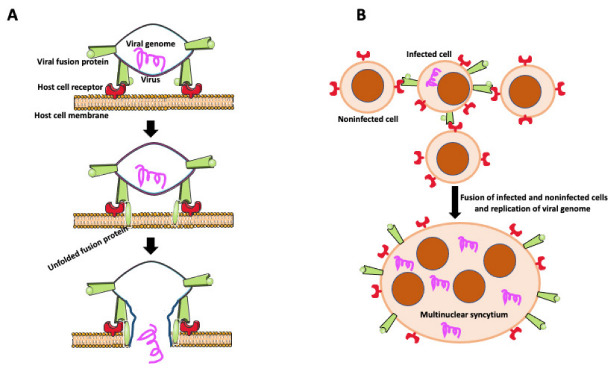
Virally induced fusion. (**A**) Fusion of the virus with the host cell membrane. Fusion of virus with the host cell membrane. Virus envelope contains fusion protein, which is recognized by receptors on the host cell membrane. After binding to its receptor, viral fusion protein unfolds, causing membrane scission and allowing the viral genome to enter the host cell. (**B**) Virally induced fusion of host cells. An infected cell expresses viral fusion protein on its surface. Fusion protein binds to its receptor on the surface of the noninfected cells promoting cell fusion. Resulting multinuclear syncytium replicates the viral genome, becoming the virus’s reservoir and facilitating the virus’s further spreading.

**Figure 5:**
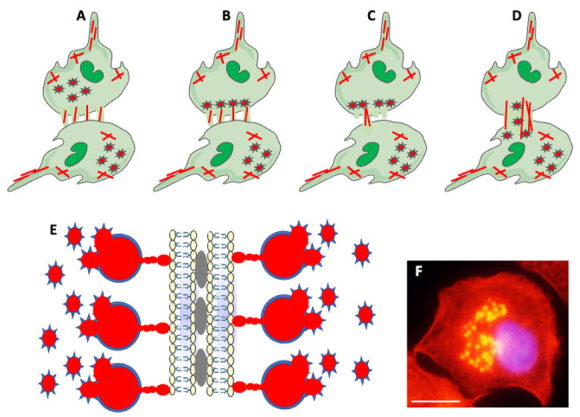
Actin-based structures in cell adhesion and fusion. (**A**) After establishing a fusion area between fusing partners, the cell-initiating fusion extends different size actin-based (red lines) protrusions at its edge. (**B**) Podosomes (stars), consisting of actin center (red) and peripheral adhesion proteins (blue), of the fusion-initiating cell migrate from the cell interior to the pre-fusion area. (**C**) One of the protrusions (usually the longest) acts as a fusopod-initiating fusion and creates the fusion pore. (**D**) Reorganization of actin filaments expands fusion pore, allowing migration of podosomes from donor to fusion partner (Modified from Faust et al., Ref. [42]). (**E**) Zipper-like structures (ZLSs) at the surface of adhering MGCs formed in response to foreign materials. Membranes of adjacent cells adhere via adhesion proteins (gray ovals). Podosomes (red and blue stars) fuse into giant actin globules (red) surrounded by adhesion proteins (blue) and attached by smaller actin globules to the membrane. Actin globules are evenly spaced (resembling the zipper) along the membrane (modified from Balbyev et al., Ref. [105]). (**F**) Image of mouse macrophage showing podosomes (yellow). The nucleus (blue) is stained with DAPI. Actin is stained red with Rhodamine-Phalloidin; podosomes look yellow because of the high actin concentration and image overexposure. The magnification bar is equal to 10 μm.

Monocyte–macrophage cell lineage derives from multipotent hematopoietic stem cells (HSCs) in the bone marrow. The classical view is that HSCs differentiate into lymphoid (LPC) and myeloid (MPC) progenitor cells. After further differentiation, LPCs generate T cells, NK cells, and B cells, while the MPCs produce basophils, eosinophils, erythrocytes, megakaryocytes, monocytes, and neutrophils. Subsequent differentiation of monocytes generates dendritic cells, macrophages, and pro-osteoclasts (Figure 1; [1,2,3,4,5]). With progress in single-cell analyses, this traditional and simplistic version of progenitor cell differentiation has been challenged. Many studies indicate that HSCs are heterogeneous and biased in their differentiation potential [6]. Studies also showed that hematopoietic stem cell bias is regulated by the distinct niche they occupy in bone marrow [7]. Based on the bias, myeloid-biased (My-Bi), balanced (Ba), lymphoid-biased (Ly-Bi), platelet-biased (Pl-Bi), and quiescent Peroxisome Proliferator-Activated Receptor γ positive (PPARγ+) osteoclast-biased (Os-Bi) progenitors have been discovered (Figure 1; [8,9,10,11,12,13,14,15,16]).

## 2. Types and Mechanisms of Fusion

Cell fusion (fusogenesis, syncytiogenesis) is widespread in natural and pathological conditions in prokaryotes and eukaryotes. It occurs, for example, during fertilization (fusion of gametes), embryogenesis (placenta/trophoblast fusion), morphogenesis, tissue development (muscle cell and osteoclast formation), tissue and organ repair, immune response, tumor development, and pathogen infection and spreading [17,18,19,20,21,22,23,24,25,26,27,28,29]. Depending on applied criteria, cell fusion can be divided into homotypic (fusion of the same cell types) versus heterotypic (different cell type fusion), and synkaryotic (homotypic or heterotypic nuclei merge creating mononuclear syncytium) versus heterokaryotic (homotypic or heterotypic multinucleated syncytium) (Figure 2A; [30,31,32,33]). Some cases fall between strict categories when fusing cells are of the same origin but at a different phase of differentiation [34,35,36]. Additionally, the origin of syncytia can differ. Usually, we reserve the term syncytium for a product of a fusion of two or more independent cells. However, a product of multiple incomplete (without or with partial cytokinesis) divisions of a single cell can also be called syncytium. Examples include nurse cell syncytia in insect ovaries, early embryonic syncytium in *Drosophila*, and ovarian germline cyst in oogenesis of *Xenopus* frog (Figure 2B; [37,38,39,40,41]).

Live imaging and video microscopy studies showed several patterns of macrophage fusion [42]: fusion between the leading edge of one cell and the cell body of another cell; and fusion of the leading edge with the posterior end of another cell or between the leading edges of both cells. The least common patterns were fusions between cell bodies and rear ends [42].

Although cell fusion mechanisms are highly diverse depending on the fusing partners and biological context, they usually require the presence of proteins mediating fusion, called fusogens. In unilateral fusion, a fusogen is present only on one of the fusing partners. In bilateral homotypic fusion, the same fusogen is present on both cells. In bilateral heterotypic fusion, fusing cells contain different fusogens [43]. Before fusion, cells must overcome an existing powerful thermodynamic repulsion of membrane lipid bilayers and make direct (~nm distance) contact [43,44,45]. Next, contacting (proximal) lipid monolayers rearrange and mix. The subsequent merger of distant monolayers creates a fusion pore. Fusogen plays a role in overcoming initial repulsion and opening and expanding fusion pore [43]. The only fusogen implicated in the fusion of myeloid cells is Syncytin. Syncytin 1 and 2 in humans and Syncytin A and B in mice derived from retroviral syncytin gene integrated during evolution into the mammalian genome [34,46,47]. Syncytin binds to its receptor Sodium-Dependent Neutral Amino Acid Transporter Type 2 (ASCT-2); [34,46].

Although the specifics of fusion depend on cell type and biological context, all fusogenic proteins must decrease the energy barrier and drive lipid bilayers’ contact, followed by bilayer rearrangements and rejoining. The syncytin 1 molecule involved in fusion in mammalian placenta contains several regions: receptor binding domain, two regions forming a disulfide bond, a furin cleavage site, a fusion peptide, heptad repeats 1 and 2, a transmembrane region, and a cytoplasmic region. Endopeptidase furin cleaves syncytin, creating surface and transmembrane subunits. The binding of syncytin to its receptor changes the structural organization of the syncytin molecule. It breaks the disulfide bonds and unfolds the fusion peptide that becomes inserted into the membrane. The fusion peptide penetrates the lipid bilayer of the fusing partner. It also reorganizes actin filaments underlying membranes, which regulate the stiffness of fusing membranes. Rupture of the membrane creates a fusion pore connecting the cytoplasm of fusing cells. Rupture of the membrane creates a fusion pore connecting the cytoplasm of fusing cells. The final step consists of positional changes of heptad repeat 1 and 2 domains, membrane apposition, and bending [46].

## 3. Examples of Monocyte–Macrophage Lineage Cell Fusion

Cells of monocyte–macrophage lineage are highly syncyciogenic (fusogenic) under physiological and pathological conditions, forming syncytial multinucleated giant cells (MGCs). Examples of homotypic syncytia derived from monocyte–macrophage lineage cell fusion are osteoclasts (OCs), MGCs associated with areas of infection/inflammation called granulomas [48], and foreign body-induced giant cells (FBGCs) [34]. Additionally, monocyte–macrophages can fuse with cells of different origins, such as hepatocytes [49], T cells [50], and various circulating and tissue-resident tumor cells [51,52,53], resulting in heterotypic syncytia.

### 3.1. Osteoclasts (OCs)

Osteoclasts are bone-resorbing cells, which, together with bone-forming osteoblasts, orchestrate bone remodeling [54,55,56,57,58]. Monocyte–macrophage lineage cells differentiate and fuse into osteoclasts through the activity of the receptor activator of nuclear factor-kappa-Β ligand (RANKL) and its receptor (RANK) signaling pathway (Figure 3; [54]). Osteoclast activation is also promoted by the RANKL pathway [59]. Mature osteoclasts are large (~100 μm) cells with up to 20 nuclei [60,61]. Studies showed that giant multinuclear osteoclasts have much higher bone-resorbing activity than small osteoclasts [59,62,63,64].

### 3.2. Langhans Giant Cells and Foreign Body Induced Giant Cells (FBGCs)

Langhans giant cells (LGCs) (not to be confused with Langerhans cells) first described in tuberculosis [65] are MGCs containing a characteristic horseshoe-shaped ring of nuclei. LGCs are present within every form of granuloma, regardless of infectious or non-infectious etiology [66,67,68,69]. It is believed that LGCs play a role in restricting the pathogen/compound within the host. [69]. Foreign body-induced MGCs (FBGCs) form in response to large organic and inorganic compounds and surgical implants [67,70,71]. FBGCs are specifically adapted for phagocytosis and removal of large (above 45 μm diameter) particles, which individual macrophages cannot eliminate [67].

## 4. Tumor-Associated Macrophages (TAMs) and Cell Fusion in Cancer

Tumor-associated macrophages (TAMs) are a significant component of tumors, accounting for 30–50% of a tumor mass. In many cancers, a high density of TAMs correlates with poor patient prognosis and survival. There are two primary sources of TAMs. One is the reprogramming of tissue-resident macrophages to TAMs by the tumor milieu. Another is the recruitment of circulating monocytes into the tumor, where they differentiate into TAMs [72,73,74]. One of the theories of tumor progression and metastasis states that TAMs and other cells of myeloid origin fuse with cancer cells, allowing them to acquire motility and metastasize [52,53,75,76,77]. Macrophage fusion in breast cancer was recapitulated in cell culture. Shabo et al. [52] observed spontaneous fusion between M2 macrophages and GFP-labeled MCF-7 cancer cells. Hybrid cancer cells expressed macrophage-specific antigen CD163, which correlates with poor survival in cancer patients. The same group [53] suggested that the formation of multinucleated fusion hybrids salvages the loss of gene function/DNA damage caused by chemotherapy or radiation, allowing hybrid cells to survive and metastasize. Pavelek et al. [76] showed that cancer cells acquire macrophage molecules and pathways regulating adhesion, extracellular matrix, formation of blood vessels, chemotaxis and motility, immune response, and multidrug resistance. For example, macrophage–tumor cell hybrids express Β1,6-branched N-glycans, used by macrophages for migration. In many human cancers, expression of Β1,6-branched oligosaccharides correlates with metastasis and poor patient outcome. Authors suggest that Β1,6-branched oligosaccharides can be used as a marker of macrophage–cancer cell fusion and lead to novel therapies [76]. Seyfried and Huysentruyt [77] proposed that metastatic cancers stem from the fusion of cancer cells with myeloid cell lineage descendants, e.g., macrophages, dendritic cells, or lymphocytes with damaged mitochondria-deficient respiration caused by chronic inflammation microenvironment. Many hybrid cells express aerobic glycolysis (Warburg effect), a common feature of metastatic cancers in humans [77].

## 5. Virally Induced MGCs

Transfer of viruses between cells usually occurs by releasing viral particles from infected cells to an acellular environment and attaching to and entering uninfected cells. However, viruses enveloped by an external lipid bilayer, such as HIV-1, SARS-CoV-2, viruses from the *Herpesviridae* family, and some non-enveloped *Reoviridaeviruses* developed an additional efficient way of dissemination through direct cell-to-cell transmission. Intercellular transfer of virus may occur through intercellular projections such as tunneling nanotubes (TNTs) [46,78,79,80] or involve fusion of infected and target cells to form giant multinucleated syncytial cells [21]. In some cases, syncytial MGCs contain no more than ten nuclei (small MGCs), but they are also giant syncytia with hundreds of nuclei [21]. Infected cells express on their surface virus-encoded fusogenic proteins, which interact with receptors or surface molecules present on uninfected cells, promoting fusion (Figure 4) [21,81,82,83,84,85]. Intercellular transfer through cell fusion allows faster dissemination and evasion of the immune system. It also allows for infection of myeloid cells (dendritic cells, macrophages) naturally resistant to infection with a cell-free HIV-1 virus [86]. Myeloid cells express a high level of the sterile alpha motif and HD-domain-containing protein 1 (SAMHD1) enzyme that cleaves dNTPs necessary for viral replication [86]. Thus, the HIV-1 virus found another way to effectively disseminate and establish a virus reservoir in host tissues by fusing macrophages with infected T cells. Studies by Bracq et al. [50] detailed consecutive steps of macrophage-T cell fusion. In the first step, the infected T cell establishes contact and fuses with the uninfected macrophage. In the second step, T cell-macrophage heterotypic syncytium fuses with one or more surrounding uninfected macrophages, creating an infected MGC that survives for a long time as a reservoir of virus [50].

Many in vitro studies showed that cultured T cells infected with HIV virus form giant multinuclear syncytia. However, these in vitro observations are not necessarily true in the in vivo situation. The most thorough and realizable description of syncytia formation during HIV-1 infection in vivo comes from the studies in humanized mice harboring human lymphoid tissues. Intravital imaging of viruses encoding fluorescent tags allows for real-time tracking of syncytia formation. These studies showed that infected T cells elongate and fuse into small snake-like syncytia containing a low number of nuclei, which over time increase in size. Around 20% of all T cells formed those small syncytia. Importantly, these syncytia were mobile and disseminated the virus through transient contacts with noninfected lymphocytes [87,88].

## 6. Actin Cytoskeleton Role in Cell Fusion

Cell fusion is a multistep process involving the acquisition of fusion competence, cell movement, adhesion to the substrate, interaction between fusing partners, and eventually, the fusion of cell membranes [43]. These steps require a profound rearrangement of the cell cytoskeleton, especially actin filaments, orchestrated by small GTPases Rac-1 and RhoA pathways [42,64,70,89,90,91]. Before fusion, cells must adhere to place their membranes in close contact. Adhesion proteins such as cadherins, β2 integrin, and integrin αvβ3 participate in MGC and osteoclast adhesion. Subsequently, integrins mediate the rearrangement of the cell cytoskeleton through activation of the Rac-1 pathway [92]. Studies of the fusion of different cell types (macrophages, osteoclasts, muscle cells) in invertebrate and vertebrate animals showed the presence of (short or long) protrusion(s), sometimes called fusopodes [93] emanating from the cell edge [94,95,96,97,98,99,100,101]. Fusopods contain bands of actin filaments, and their formation is regulated by Rac-1 [93], Wiskott–Aldrich syndrome protein (WASp) family, and Arp2/3 complex, which nucleate and branch actin filaments [42,102,103]. Some studies of myeloid cell fusion indicate that tunneling nanotubes (TNTs), which contain actin filaments and/or microtubules [79], can also function as fusopodes [34].

Besides cell extensions, other actin-based structures participating in cell fusion are podosomes and podosome-derived zipper-like structures (Figure 5). Podosomes are actin-rich membrane protrusions containing a core of branched F-actin and actin-regulatory proteins surrounded by an adhesion ring of integrins, vinculin, and talin. Podosomes play a role in stabilizing cell extensions, sensing rigidity and topography of milieu, adhesion to the substrate, and extracellular matrix degradation [34,104]. Zipper-like structures (ZLSs), containing periodic bands of actin resembling zipper, are involved in cell-to-cell interactions and bridging two cell membranes [105,106,107]. Studies by Balbyev et al. [105] showed that ZLSs present at the surface of adhering MGCs formed in response to foreign materials are temporary structures lasting about 15 min. They derive from podosomes and reconstitute into podosomes. Besides actin, ZLSs also contain adhesion proteins typical for podosomes. Authors suggest that ZLSs bridge “zippered up” membranes of MGCs but do not function in cell fusion per se (Figure 5 [105]). In contrast, ZLSs described by Takito et al. [106] in osteoclasts participate in the cell fusion process. However, the osteoclasts’ ZLSs do not derive from podosomes, do not contain adhesion proteins, and form through a continuous retrograde flow of actin [107]. Thus, ZLSs of MGCs and osteoclasts share similar morphology but have different functions. Faust et al. [42] studied, in detail, the relationship between fusogenic protrusions and podosomes in fusing macrophages. They observed that, before the fusion, a wave of podosomes migrates from the macrophage interior to the periphery. Subsequently, podosomes align along the cell membrane of the impending fusion area enriched in extending/retracting cell protrusions. Soon after aligning podosomes, one protrusion initiates fusion. Following fusion pore formation, actin filaments reorganize, expanding the pore, and podosomes translocate from the donor cell to the fusion partner (Figure 5 [42]).

During cell fusion, fusing partners must remodel their cytoplasmic membranes. Remodeling requires assembly and disassembly of cortical actin filament bundles underlying the membranes. Recent studies showed that cullin 3-based E3 ubiquitin ligase CUL3^KCTD10^ controls the reorganization of cortical actin in fusing myoblasts. Cortical actin bundles are stabilized at contacting cell membranes by EPS8–IRSp53 complexes. These complexes are also known to activate the Rac-1 pathway and regulate filopodia formation, cancer cell motility, and metastasis [108,109]. Monoubiquitylation of EPS8 by CUL3^KCTD10^ removes EPS8–IRSp53 from the membrane cortex, preventing actin bundling and allowing membrane fusion [110]. Although this process has been described in myoblast fusion, ubiquitination may be a universal mechanism controlling fusion in other cell types.

Although the molecular components of the signaling pathways involved in macrophage–monocyte lineage cell fusion are well-characterized (see other chapters in this volume), further studies are needed to establish the functional relationship and causality between actin filaments, podosomes, ZLSs, fusopodes, and fusogenic proteins during the cell fusion process.

## Data Availability

Not applicable.
